# Assessing stability of gene selection in microarray data analysis

**DOI:** 10.1186/1471-2105-7-50

**Published:** 2006-02-01

**Authors:** Xing Qiu, Yuanhui Xiao, Alexander Gordon, Andrei Yakovlev

**Affiliations:** 1 Department of Biostatistics and Computational Biology, University of Rochester, 601 Elmwood Avenue, Rochester, New York 14642, USA

## Abstract

**Background:**

The number of genes declared differentially expressed is a random variable and its variability can be assessed by resampling techniques. Another important stability indicator is the frequency with which a given gene is selected across subsamples. We have conducted studies to assess stability and some other properties of several gene selection procedures with biological and simulated data.

**Results:**

Using resampling techniques we have found that some genes are selected much less frequently (across sub-samples) than other genes with the same adjusted *p*-values. The extent to which this type of instability manifests itself can be assessed by a method introduced in this paper. The effect of correlation between gene expression levels on the performance of multiple testing procedures is studied by computer simulations.

**Conclusion:**

Resampling represents a tool for reducing the set of initially selected genes to those with a sufficiently high selection frequency. Using resampling techniques it is also possible to assess variability of different performance indicators. Stability properties of several multiple testing procedures are described at length in the present paper.

## Background

The result of every analysis of microarray data is an outcome of a random experiment. For example, the number of genes declared differentially expressed and the estimated false discovery rate (FDR) should be treated as random variables and their variability has to be assessed in the same fashion that the population variance is estimated in the usual statistical inference. The variance of the number of differentially expressed genes (as well as other outcomes of a given selection procedure) may depend on the chosen statistical test, method of multiple testing adjustment, effect sizes for different genes, and the correlation structure of the data. The latter factor deserves special attention. Although some normalization procedures may lead to a significant reduction in the correlation between gene expression levels, and thus between the associated test statistics, the remaining correlation may be strong enough to have a disastrous effect on the statistical inference from microarray data [1]. The effect correlations in microarray data on variability of the most basic performance indicators of various testing procedures calls for further investigation.

There is another facet of the problem to consider. Every specific analysis of microarray data results in a list of candidate genes that are deemed differentially expressed across the two conditions under study. The composition of this list is subject to random fluctuations and this effect also needs to be quantitatively assessed. Even if one concentrates on the selection of individual genes rather than gene combinations, the situation here is similar to that in the regression analysis aimed at selecting significant explanatory variables (covariates). When focusing on a specific variable, one can observe a certain degree of instability of this variable selection inherent in any pertinent statistical procedure. The term "stability" means "replication stability" for the selection of significant variables. This kind of stability is easy to assess and interpret in simulation studies where the "true" set of differentially expressed genes is known. When analyzing biological data, one can resort to resampling techniques for this purpose [2]. In particular, one can apply a subsampling counterpart of the "delete-*d*-jackknife" procedure to the sample at hand and estimate the frequency with which a given gene has been selected across all sub-samples. Then an additional selection criterion can be imposed by finally selecting only those genes with a frequency of selection greater than, say, 80%.

The above discussion suggests the following two ways of using resampling techniques in microarray data analysis. These techniques can be used to assess stability characteristics of a given selection procedure and compare different procedures. In this case, one is usually interested in certain characteristics of the whole set of selected genes rather than its individual members. In their very interesting paper, Pavlidis et al. [3] used leave-one-out resampling to study the stability of gene selection in conjunction of the required number of replicates in the analysis of differential expression of genes. The authors proposed two stability measures (metrics) to compare the ranked list of the genes originally selected to the ranking obtained when one replicate is removed. Then the stability measures are averaged over the subsamples. The first measure refers to the fraction of genes among the originally selected ones that are recovered in a given subsample. Stolovitzky [4] proposed a similar measure which is not conditioned on the set of genes originally selected from the data prior to resampling. However, statistical properties of the robustness index introduced by Stolovitzky remain unclear. The second measure by Pavlidis et al. [3] is more subtle; it has to do with the degree to which the ordering is preserved and can be used whenever the number of selected genes does not show strong variations among subsamples. We propose the delete-*d*-jackknife variance of the number of selected genes (which is the primary endpoint to be assessed when comparing different methodologies) across subsamples as a pertinent measure of stability of a chosen testing procedure. This measure has clear statistical properties and is easier to interpret. The distribution of the number of selected genes can also be estimated using the delete-*d*-jackknife method [5]. Another way of using resampling techniques is to assess the stability of selection for individual genes in line with the currently practiced methodology of significance testing in microarray analysis. This can be accomplished by estimating the frequency of selection of each gene given it has been selected at least in one subsample. As we show in the present paper, this measure also provides valuable information on the performance of each selection procedure when its dependence on adjusted *p*-values is included in the analysis.

We have conducted a simulation study to evaluate the effect of correlation between gene expression levels on the performance of several selection procedures in terms of the variability of such important indicators as the number of selected genes and the proportion of falsely rejected among all rejected null hypotheses. All these indicators are directly accessible in computer simulations, thereby providing an explanatory insight into the performance of different procedures. From this perspective, the Bonferroni and Westfall-Young multiple testing procedures are explored in conjunction with the Student *t*, Kolmogorov-Smirnov, and Cramér-von Mises two-sample tests. The latter two tests are distribution free. The Bonferroni and Westfall-Young step-down procedures [6] are designed to control the familywise error rate (FWER). The FWER is defined loosely as the probability of at least one Type 1 error in the context of multiple testing. The FDR-based procedures are also explored; these are represented by the empirical Bayes method [7-9] as well as the Benjamini-Hochberg and Benjamini-Yakutieli procedures [10,11]. The FDR is defined as the expected fraction of falsely rejected among all rejected hypotheses. It should be noted that our simulation studies do not attempt to model the actual correlation structure of microarray data; their only purpose is to see which specific performance indicators may be sensitive to the presence of correlation in the data. The quantitative characteristics we report from the simulated data cannot be extrapolated to biological data and can only be viewed as proof of principle.

Another set of experiments was concerned with actual biological data. We assessed probabilistic characteristics of the number of selected genes by resampling from a large set of data on two types of childhood leukemia available from the St. Jude Children's Research Hospital Database [12]. Using this data set, we also assessed the replication stability of gene selection and its dependence on adjusted *p*-values.

## Methods

### Biological data

For the purposes of this study, use was made of the St. Jude Children's Research Hospital (SJCRH) Database on childhood leukemia which is publicly available on their website under the Supplemental Data section: [12] The whole SJCRH Database contains gene expression data on 335 subjects, each represented by a separate array (Affymetrix, Santa Clara, CA) reporting measurements on the same set of 12558 genes. We selected two groups of patients with hyperdiploid (Hyperdip) and T-cell acute lymphoblastic leukemia (TALL), respectively. The groups were balanced to include 43 patients in each group. Since the nature of our study was purely methodological, the choice of the data set was quite arbitrary; it was dictated solely by sample size considerations. The microarray data thus chosen were background corrected and normalized using the Bioconductor RMA software. This software implements the quantile normalization procedure [13,14] carried out at the probe feature level. After the normalization, each gene is represented in the final data set by the logarithm (base 2) of its expression level.

### Simulated data

Our simulation study was designed to illustrate the effect of correlation on the performance of gene selection procedures. We simulated 2*n *independent multi-variate normal random vectors with exchangeable correlation structure, each representing log-intensities of 1255 genes of which the first 125 genes were designated to be differentially expressed. Two sets of simulations were conducted with the sample size chosen to be *n *= 15 and *n *= 43, respectively. We use the following self-explanatory notation for the four sets of simulated data: SIM15, SIM15CORR, SIM43, SIM43CORR. In total, 200 independent data sets, each consisting of 2*n *simulated vectors, were generated for each sample size. The marginal distributions of the log-intensities of "Not Different" genes were standard normal, while the log-intensities of "Different" genes expressions followed the normal distribution with mean two and unit variance.

The exchangeable pairwise correlation structure was superimposed on the normal vectors with independent components as discussed in [1]. Briefly, we first generate a 1255 × 2*n *matrix with each entry being an independent realization of a standard normal random variable. To model a set of "Different" genes, we add a value of 2 to the first 125 rows in the first group and denote the resultant matrix by *X *= {*x*_*ij*_}, *i *= 1, ..., 1255; *j *= 1, ..., 2*n*. All the elements *xij *of this matrix are stochastically independent, but those with *i *= 1, 2, ..., 125 and *j *= 1, 2, ..., *n *are normally distributed with mean 2 and unit variance. Expression levels of the genes outside this special set of 125 genes follow the standard normal distribution. Next we generate a 2*n*-dimensional random vector with independent and identically distributed components, each component having a standard normal distribution. Denote this vector by *A *= {*a*_*j*_}, *j *= 1, ..., 2*n*. Define yij=ρaj+1−ρxij
 MathType@MTEF@5@5@+=feaafiart1ev1aaatCvAUfKttLearuWrP9MDH5MBPbIqV92AaeXatLxBI9gBaebbnrfifHhDYfgasaacH8akY=wiFfYdH8Gipec8Eeeu0xXdbba9frFj0=OqFfea0dXdd9vqai=hGuQ8kuc9pgc9s8qqaq=dirpe0xb9q8qiLsFr0=vr0=vr0dc8meaabaqaciaacaGaaeqabaqabeGadaaakeaacqWG5bqEdaWgaaWcbaGaemyAaKMaemOAaOgabeaakiabg2da9maakaaabaacciGae8xWdihaleqaaOGaemyyae2aaSbaaSqaaiabdQgaQbqabaGccqGHRaWkdaGcaaqaaiabigdaXiabgkHiTiab=f8aYbWcbeaakiabdIha4naaBaaaleaacqWGPbqAcqWGQbGAaeqaaaaa@3FE1@, *i *= 1, ..., 1255; *j *= 1, ..., 2*n*, so that for any *i*_1 _≠ *i*_2 _and *j *we have corr (yi1j
 MathType@MTEF@5@5@+=feaafiart1ev1aaatCvAUfKttLearuWrP9MDH5MBPbIqV92AaeXatLxBI9gBaebbnrfifHhDYfgasaacH8akY=wiFfYdH8Gipec8Eeeu0xXdbba9frFj0=OqFfea0dXdd9vqai=hGuQ8kuc9pgc9s8qqaq=dirpe0xb9q8qiLsFr0=vr0=vr0dc8meaabaqaciaacaGaaeqabaqabeGadaaakeaacqWG5bqEdaWgaaWcbaGaemyAaK2aaSbaaWqaaiabigdaXaqabaWccqWGQbGAaeqaaaaa@3233@, yi2j
 MathType@MTEF@5@5@+=feaafiart1ev1aaatCvAUfKttLearuWrP9MDH5MBPbIqV92AaeXatLxBI9gBaebbnrfifHhDYfgasaacH8akY=wiFfYdH8Gipec8Eeeu0xXdbba9frFj0=OqFfea0dXdd9vqai=hGuQ8kuc9pgc9s8qqaq=dirpe0xb9q8qiLsFr0=vr0=vr0dc8meaabaqaciaacaGaaeqabaqabeGadaaakeaacqWG5bqEdaWgaaWcbaGaemyAaK2aaSbaaWqaaiabikdaYaqabaWccqWGQbGAaeqaaaaa@3235@) = *ρ *In the present study, the correlated data were generated for a single value of the correlation coefficient *ρ *= 0.6. This high correlation coeffcient was chosen to more clearly demonstrate the effects of correlation. However, this value is not overly unrealistic because the mean (over all gene pairs) correlation coefficient estimated from the raw data referred to in Section 2.1 is equal to 0.72.

In order to see whether or not the stability of gene selection is related to the power, our explanatory simulations were conducted under two different scenarios. Under the first scenario, the sample size was small (*n *= 15) so that the power was lower than 100%. Under the second scenario the sample size was sufficiently large (*n *= 43) to attain a 100% power.

### Resampling techniques

When analyzing biological data, we used a subsampling version of the delete-*d *jackknife method [5,15,16], which is technically equivalent to the leave-*d*-out cross-validation. It can be proven that if n
 MathType@MTEF@5@5@+=feaafiart1ev1aaatCvAUfKttLearuWrP9MDH5MBPbIqV92AaeXatLxBI9gBaebbnrfifHhDYfgasaacH8akY=wiFfYdH8Gipec8Eeeu0xXdbba9frFj0=OqFfea0dXdd9vqai=hGuQ8kuc9pgc9s8qqaq=dirpe0xb9q8qiLsFr0=vr0=vr0dc8meaabaqaciaacaGaaeqabaqabeGadaaakeaadaGcaaqaaiabd6gaUbWcbeaaaaa@2E2C@/*d *→ 0 and *n *- *d *→ ∞, then the delete-*d*-jackknife is consistent for the median [15]. Therefore, the general recommendation is to leave out more than *d *= n
 MathType@MTEF@5@5@+=feaafiart1ev1aaatCvAUfKttLearuWrP9MDH5MBPbIqV92AaeXatLxBI9gBaebbnrfifHhDYfgasaacH8akY=wiFfYdH8Gipec8Eeeu0xXdbba9frFj0=OqFfea0dXdd9vqai=hGuQ8kuc9pgc9s8qqaq=dirpe0xb9q8qiLsFr0=vr0=vr0dc8meaabaqaciaacaGaaeqabaqabeGadaaakeaadaGcaaqaaiabd6gaUbWcbeaaaaa@2E2C@ but much fewer that *n *observations. A similar recommendation holds for the variance [15,16]. We used *d *= 7 to perturb the data set, which is only slightly greater than n
 MathType@MTEF@5@5@+=feaafiart1ev1aaatCvAUfKttLearuWrP9MDH5MBPbIqV92AaeXatLxBI9gBaebbnrfifHhDYfgasaacH8akY=wiFfYdH8Gipec8Eeeu0xXdbba9frFj0=OqFfea0dXdd9vqai=hGuQ8kuc9pgc9s8qqaq=dirpe0xb9q8qiLsFr0=vr0=vr0dc8meaabaqaciaacaGaaeqabaqabeGadaaakeaadaGcaaqaaiabd6gaUbWcbeaaaaa@2E2C@, to be as close as possible to the most widely used delete-one version of jackknife. It should be noted that the delete-1-jackknife method may be inconsistent for some estimators (sample quantiles representing a typical example) and the delete-*d*-jackknife was proposed to remedy this problem [16]. When implemeting the delete-*d*-jackknife method, we resorted to sampling without replacement because the empirical Bayes method is very sensitive to ties. As far as subsampling versions of the delete-*d*-jackknife method are concerned, the schemes with and without replacement are essentially identical when the number of subsamples is large [16].

The total number of subsamples was typically equal to 200. In a separate study, we ascertained that the results for 1000 subsamples were largely similar. Let *Z *be the number of selected genes. The variance of *Z *is estimated by a resampling counterpart of the jackknife sample variance [16]:

V=n−ddB∑l=1B(Zn−d,l−1B∑k=1BZn−d,k)2,
 MathType@MTEF@5@5@+=feaafiart1ev1aaatCvAUfKttLearuWrP9MDH5MBPbIqV92AaeXatLxBI9gBaebbnrfifHhDYfgasaacH8akY=wiFfYdH8Gipec8Eeeu0xXdbba9frFj0=OqFfea0dXdd9vqai=hGuQ8kuc9pgc9s8qqaq=dirpe0xb9q8qiLsFr0=vr0=vr0dc8meaabaqaciaacaGaaeqabaqabeGadaaakeaacqWGwbGvcqGH9aqpdaWcaaqaaiabd6gaUjabgkHiTiabdsgaKbqaaiabdsgaKjabdkeacbaadaaeWbqaamaabmaabaGaemOwaO1aaSbaaSqaaiabd6gaUjabgkHiTiabdsgaKjabcYcaSiabdYgaSbqabaGccqGHsisldaWcaaqaaiabigdaXaqaaiabdkeacbaadaaeWbqaaiabdQfaAnaaBaaaleaacqWGUbGBcqGHsislcqWGKbazcqGGSaalcqWGRbWAaeqaaaqaaiabdUgaRjabg2da9iabigdaXaqaaiabdkeacbqdcqGHris5aaGccaGLOaGaayzkaaaaleaacqWGSbaBcqGH9aqpcqaIXaqmaeaacqWGcbGqa0GaeyyeIuoakmaaCaaaleqabaGaeGOmaidaaOGaeiilaWcaaa@5779@

where *B *is the total number of subsamples (*B *= 200), *Z*_*n*-*d*, *j *_is the statistic *Z *evaluated at the *j*th delete-*d *jackknife subsample. The variance of the number of selected genes was used as a criterion of stability of the testing procedures under study. The corresponding distributions were also estimated. Another criterion was the selection stability for each individual gene measured by the frequency of selection conditional on the event of selection in at least one of the subsamples.

### Selection of differentially expressed genes

When resorting to the Bonferroni adjustment, one needs to compute unadjusted *p*-values from the sampling distribution of the test statistic under consideration. For the *t*-test we used quantiles of the Student distribution. Among the distribution-free methods, the Cramér-von Mises test [17] represents an appealing alternative to the Kolmogorov-Smirnov test. The reason is that the granularity of the Cramér-von Mises statistic (which causes granularity of the corresponding *p*-values) is much smaller than that for the Kolmogorov-Smirnov test. As a result, the *p*-values corresponding to the critical region increase much more steeply for the Kolmogorov-Smirnov test than for the Cramér-von Mises test, thereby making the Kolmogorov-Smirnov test less powerful.

To describe the Cramér-von Mises test, consider two independent samples *x*_1_, *x*_2_, ..., *x*_*m *_and *y*_1_, *y*_2_, ..., *y*_*n *_from distributions *F*(*x*) and *G*(*x*), respectively, and let *F*_*m *_and *G*_*n *_be their respective empirical distribution functions. We wish to test the following null hypothesis **H**_0_:*F*(*x*) = *G*(*x*) for all *x *versus the alternative: *F *≠ *G*. The Cramér-von Mises test-statistic for the hypothesis **H**_0 _is defined as the (squared) *L*_2 _distance between *F*_*m*_(*x*) and *G*_*n*_(*x*):

W2=mn(m+n)2{∑i=1m[Fm(xi)−Gn(xi)]2+∑j=1n[Fm(yj)−Gn(yj)]2}.
 MathType@MTEF@5@5@+=feaafiart1ev1aaatCvAUfKttLearuWrP9MDH5MBPbIqV92AaeXatLxBI9gBaebbnrfifHhDYfgasaacH8akY=wiFfYdH8Gipec8Eeeu0xXdbba9frFj0=OqFfea0dXdd9vqai=hGuQ8kuc9pgc9s8qqaq=dirpe0xb9q8qiLsFr0=vr0=vr0dc8meaabaqaciaacaGaaeqabaqabeGadaaakeaacqWGxbWvdaahaaWcbeqaaiabikdaYaaakiabg2da9maalaaabaGaemyBa0MaemOBa4gabaGaeiikaGIaemyBa0Maey4kaSIaemOBa4MaeiykaKYaaWbaaSqabeaacqaIYaGmaaaaaOWaaiWaaeaadaaeWbqaaiabcUfaBjabdAeagnaaBaaaleaacqWGTbqBaeqaaOGaeiikaGIaemiEaG3aaSbaaSqaaiabdMgaPbqabaGccqGGPaqkcqGHsislcqWGhbWrdaWgaaWcbaGaemOBa4gabeaakiabcIcaOiabdIha4naaBaaaleaacqWGPbqAaeqaaOGaeiykaKIaeiyxa01aaWbaaSqabeaacqaIYaGmaaGccqGHRaWkdaaeWbqaaiabcUfaBjabdAeagnaaBaaaleaacqWGTbqBaeqaaOGaeiikaGIaemyEaK3aaSbaaSqaaiabdQgaQbqabaGccqGGPaqkcqGHsislcqWGhbWrdaWgaaWcbaGaemOBa4gabeaakiabcIcaOiabdMha5naaBaaaleaacqWGQbGAaeqaaOGaeiykaKIaeiyxa01aaWbaaSqabeaacqaIYaGmaaaabaGaemOAaOMaeyypa0JaeGymaedabaGaemOBa4ganiabggHiLdaaleaacqWGPbqAcqGH9aqpcqaIXaqmaeaacqWGTbqBa0GaeyyeIuoaaOGaay5Eaiaaw2haaiabc6caUaaa@722F@

The asymptotic theory for the Cramér-von Mises test was developed by Anderson and Darling [18], Rosenblatt [19], Anderson [20], and Csorgo and Faraway [21]. The asymptotic approximation of the distribution of *W*^2 ^under **H**_0 _is of little utility in microarray data analysis because it is very inaccurate whenever one works with extremely small *p*-values required by the FWER controlling multiple testing procedures. For example, when *n *= *m *= 43 and the exact *p*-value for the Cramér-von Mises test is equal to 3.928 × 10^-6^, its asymptotic approximation gives 6.897 × 10^-6^, a much larger *p*-value. The above-mentioned exact *p*-value of 3.928 × 10^-6^corresponds to the adjusted (by the Bonferroni method) *p*-value of about 0.049 when testing 12558 hypotheses as in our application described in Section 3.1. Therefore, one needs an algorithm for computing exact quantiles of the Cramér-von Mises sampling distribution. We used the method proposed by Burr [22] for this purpose. It should be noted that the needed small *p*-values for the Cramér-von Mises test cannot be estimated with sufficient accuracy by permuting the test-statistics, because the required number of permuations is astronomical [23] and cannot be accomplished with present-day hardware.

The Westfall-Young step-down algorithm [6] bypasses the stage of computing unadjusted *p*-values and goes directly to the estimation of adjusted *p*-values at a given level of the FWER. We carried out 10,000 permutations to model a null distribution of each test statistic. We also used the multiple testing adjustment proposed by Benjamini and Hochberg [10] and its modification by Benjamini and Yekutieli [11]. The more conservative Benjamini and Yekutieli procedure is warranted with normalized data because the quantile normalization is known to induce negative correlations in microarray data [1]. The nonparametric empirical Bayes method by Efron et al. [7-9] was one more method of choice in the present paper. We used kernel smoothing (with the Gaussian kernel) for density estimation to implement the empirical Bayes method. The threshold level of the posterior probability was set at 0.95.

To distinguish between different statistical procedures, we use the following notation:

B/t – *t*-test with Bonferroni adjustment;

B/KS – Kolmogorov-Smirnov test with Bonferroni adjustment;

B/CVM – Cramér-von Mises test with Bonferroni adjustment;

WY/t – *t*-test with Westfall-Young algorithm;

WY/KS – Kolmogorov-Smirnov test with Westfall-Young algorithm;

WY/CVM – Cramér-von Mises with Westfall-Young algorithm;

BH/t – *t*-test with Benjamini-Hochberg adjustment;

BY/t – *t*-test with Benjamini-Yekutieli adjustment;

EB/t – *t*-test with gene selection by nonparametric empirical Bayes method.

### False discovery rate and power

We provide estimates of the FDR only for simulations. We do not report FDR estimates for biological data because only indirect methods [24,25] are available in this case. Such methods introduce an additional variation in the estimates which is impossible to distinguish from that caused by a given selection procedure. In our simulation studies, the true FDR was estimated directly as the proportion of false discoveries among all discoveries. Then the sample mean (across the 200 samples) of this nonparametric estimate is reported together with the corresponding standard deviation. It happened only once (when applying the Kolmogorov-Smirnov test with Bonferroni adjustment to a sample of size *n *= 15) that we set the estimated FDR at zero (see [26] for the definition of the positive FDR). Since the expression levels of the 125 differentially expressed genes are identically distributed, the power can be defined as the expected proportion of correct discoveries among the 125 true alternative hypotheses. We provide the usual nonparametric estimates of the power thus defined and its standard deviation.

### Software

The relevant software is included in the Additional Material Files [see additional file 1].

## Results

### Analysis of biological data

Table 1 presents the results of the delete-7-jackknife subsampling applied to the selected set of biological data. In this study, the FWER is controlled at the level of 0.05. Shown in the parentheses is the percentage of "stable" genes relative to the mean (over the 200 subsamples) number of selected genes computed under the additional requirement of at least 80% occurrence in the set of selected genes. This percentage remains practically unchanged when changing the FWER control level. The standard deviation of the number of selected genes is quite high for all the procedures studied. The proportion of highly stable (with at least 80% occurrence) genes appears to be virtually the same for all the tests and multiple testing procedures. However, the situation is not the same when looking at less frequent genes as discussed below.

**Table 1:** Delete-*d*-jackknife subsampling for the biological data with *d *= 7.

Method	Leave-seven-out Jackknife		
	
	Mean number of selected genes	Standard deviation	Mean number of stable genes and its proportion to the mean of selected genes
B/KS	622	71	504(80.99%)
B/CVM	1096	123	853(77.80%)
B/t	775	103	644(83.05%)
WY/KS	685	153	533(77.82%)
WY/CVM	889	124	711(79.98%)
WY/t	876	110	726(82.89%)
EB/t	1867	438	1481(79%)
BH/t	2726	445	2176(80%)
BY/t	1599	222	1282(80%)

**Figure 1:**
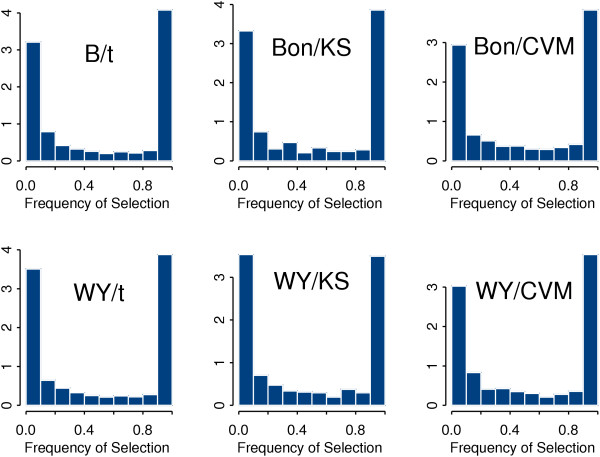
Histograms of the frequency of occurence in the set of selected genes obtained by delete-7-jackknife subsampling from the SJCRH data.

Shown in Figure 1 are the proportions of genes with different frequencies of selection among those genes that have been selected at least once in the course of delete-seven subsampling. It is seen from this figure that the histograms are *U*-shaped so that one can distinguish two extreme groups of genes characterized by high and low stability, respectively. The proportions of genes in each "intermediate-frequency" category are relatively small. This phenomenon persists for all the statistical tests under study when the FWER is controlled either by the Bonferroni adjustment or by the Westfall-Young permutation algorithm. It is clear from Figure 1 that the population of genes selected at least once across all subsamples is heterogeneous with respect to their stability characterized by the frequency of selection.

**Figure 2:**
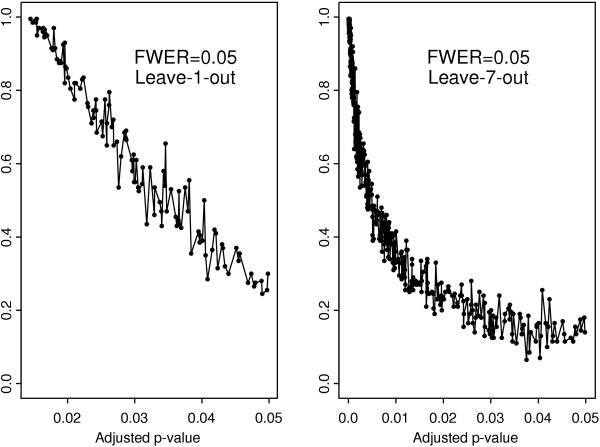
Frequency of occurrence in the set of selected genes versus adjusted *p*-values for the *t*-test with Bonferroni adjustment. Left panel: delete-1-jackknife subsampling, right panel: delete-7-jackknife subsampling.

To gain a better insight into this heterogeneity, it makes sense to look at the relationship between the frequency of occurrence and the corresponding *p*-values. To this end, we produced scatter-plots for the frequency of occurrence in the set of selected genes across the sub-samples and the original adjusted *p*-values determined by the application of each testing procedure to the whole set of arrays. The results for the *t*-test with Bonferroni adjustment are given in Figure 2. The leave-seven-out resampling reveals a non-linear (but still monotonic) pattern showing that the relationship in question may be quite complex. For comparison, we also present the result for the leave-one-out procedure, in which case the dependence appears to be almost linear but the scatter of points is wide because this procedure does not perturb the data sufficiently. In what follows, we will discuss only the observations resulted from the delete-7-jackknife subsampling.

**Figure 3:**
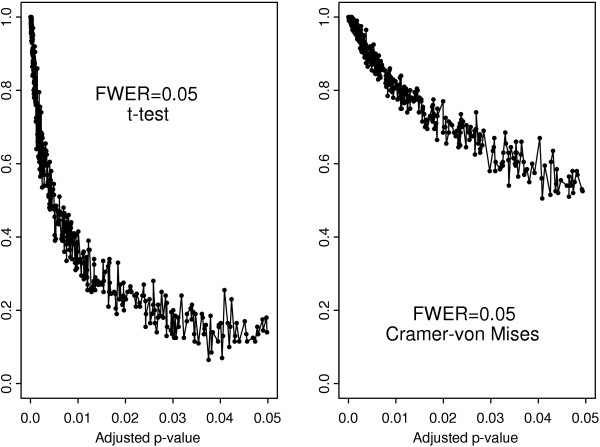
Frequency of occurrence in the set of selected genes versus adjusted *p*-values for the *t*- and Cramér-von Mises test with Bonferroni adjustment.

**Figure 4:**
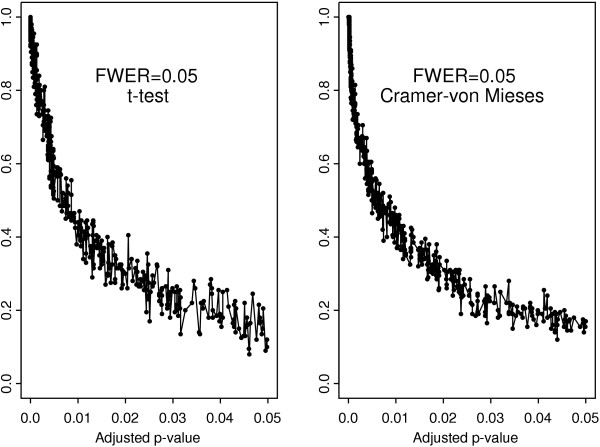
Frequency of occurrence in the set of selected genes versus adjusted *p*-values for the *t*- and Cramér-von Mises test with Westfall-Young algorithm.

**Figure 5:**
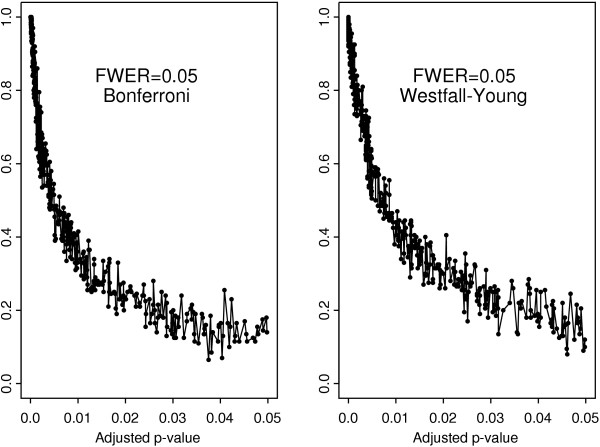
Frequency of occurrence in the set of selected genes versus adjusted *p*-value for the *t*-test with Bonferroni adjustment and Westfall-Young algorithm.

**Figure 6:**
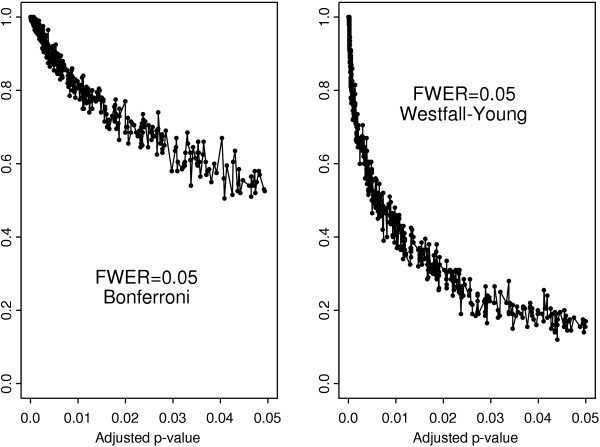
Frequency of occurrence in the set of selected genes versus adjusted *p*-values for the Cramér-von Mises test with Bonferroni adjustment and Westfall-Young algorithm.

The results for the *t*-test and the Cramér-von Mises test with Bonferroni adjustment are compared in Figure 3. It is clear that the genes selected by the Cramér-von Mises test are uniformly more stable than those selected by the *t*-test. The difference is much less pronounced with the Westfall-Young algorithm as evidenced by Figure 4. Both multiple testing procedures yield similar scatter plots for the *t*-test showing its overall poor stability in comparison to the Cramér-von Mises test (Figure 5). In contrast, the stability of the Cramér-von Mises test can be increased substantially when using the more conservative Bonferroni adjustment in place of the Westfall-Young procedure (Figure 6). These results show that the stability of gene selection provides an important additional information on each selected gene and this information can be extracted from real data by resorting to resampling techniques.

**Figure 7:**
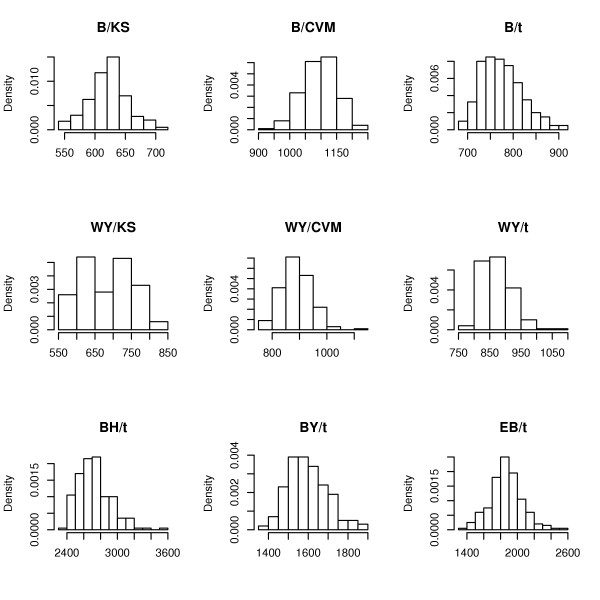
Histograms of the number of selected genes across 200 subsamples for different methods applied to the SJCRH data.

The mean values and standard deviations of the number of genes selected by different multiple testing procedures are reported in Tables 1. It is also interesting to look at the shape of the corresponding distribution. Figure 7 shows that this shape varies widely for different procedures. The nearly symmetric form of this distribution in combination with a relatively small variance is an appealing feature of the Cramér-von Mises test.

### Analysis of simulated data

To demonstrate the effect of correlation between gene expression levels on the performance of gene selection procedures, we carried out simulation studies as described in Section 2.2. Table 2 presents the most basic performance indicators for the sample size *n *= *m *= 15. Since the simulated data are normally distributed it comes as no surprise that the *t*-test proves itself as the most powerful one among those under study. With this small sample size, however, even the *t*-test tends to be underpowered when used in combination with the Bonferroni adjustment or Westfall-Young adjustments. The power of the *t*-test is much higher with the Benjamini-Hochberg and nonparametric empirical Bayes procedures. The variance of the estimated power as well as the number of selected genes increases dramatically with increasing correlation between gene expression signals.

**Table 2:** Simulating the basic characteristics of gene selection procedures, 125 differentially expressed genes, 200 simulation runs, *n *= 15. The table presents mean values over simulation runs. Standard deviations are given in parentheses.

Method	Number of Selected Genes		FDR		Power	
	
	S15	S15COR	S15	S15COR	S15	S15COR
B/KS	36(5.3)	34(18.8)	<0.0006	<0.0001	0.28(0.04)	0.27(0.15)
B/CVM	80(4.9)	80(25.0)	<0.0008	<0.0004	0.64(0.04)	0.64(0.20)
B/t	89(5.5)	88(24.7)	<0.0007	<0.0008	0.71(0.04)	0.70(0.20)
WY/KS	36(4.7)	53(26.3)	0	<0.0008	0.29(0.04)	0.43(0.21)
WY/CVM	81(5.6)	90(21.25)	<0.0003	<0.0008	0.65(0.04)	0.72(0.17)
WY/t	90(5.4)	98(19.66)	<0.0007	0.0009	0.72(0.04)	0.79(0.16)
BH/t	130(2.8)	139(73.5)	0.048(0.019)	0.051(0.135)	0.99(0.01)	0.99(0.03)
EB/t	116(3.0)	141(100.0)	0.012(0.006)	0.052(0.157)	0.92(0.02)	0.96(0.07)

Table 3 shows the results for a larger sample size (*n *= *m *= 43). In this case, all the methods attain 100% power. For all the FWER controlling procedures, the mean number of selected genes is exactly 125 and the corresponding variance is quite small irrespective of the presence or absence of correlation between gene expression levels. The FDR estimates are also uniformly small for such procedures as indicated by Table 3. However, the effect of correlation on the standard deviation of the number of selected genes is still very strong (compare with Table 2) for the Benjamini-Hochberg and nonparametric empirical Bayes procedures, indicating the inherent instability of these procedures. It should be noted that there is also a dramatic effect of the correlation on the standard deviation of the FDR observed for the latter procedures (Table 3). The results for 1000 simulation runs were largely similar.

**Table 3:** Simulating the basic characteristics of gene selection procedures, 125 differentially expressed genes, 200 simulation runs, *n *= 43. The table presents mean values over simulation runs. Standard deviations are given in parentheses.

Method	Number of Selected Genes		FDR		Power	
	
	SIM43	SIM43CORR	SIM43	SIM43CORR	SIM43	SIM43CORR
B/KS	125(0.3)	125(0.5)	<0.0003	<0.0001	1	1
B/CVM	125(0.3)	125(0.3)	<0.0005	<0.0005	1	1
B/t	125(0.2)	125(0.4)	<0.0003	<0.0006	1	1
WY/KS	125(0.4)	125(0.4)	<0.0002	<0.0003	1	1
WY/CVM	125(0.3)	125(0.4)	<0.0006	<0.0010	1	1
WY/t	125(0.2)	125(0.3)	<0.0003	<0.0008	1	1
BH/t	131(2.7)	140(90.0)	0.0427(0.0192)	0.0356(0.1219)	1	1
EB/t	125(0.2)	133(60.0)	0.0082(0.0012)	0.0246(0.1024)	1	1

We also include the histograms for the number of selected genes resulted from our simulation studies [see additional file 2]. Note that the high variance observed for the BH/t and EB/t procedures (Figures 2 and 4 in the Additional File 2) is mainly attributable to outliers.

## Discussion

Numerous publications have considered the utility of multiple testing procedures in the context of microarray data analysis (see [27] for a review). However, little attention has been given to the replication stability of such procedures which is related to the reproducibility of scientific results. Our study shows that the variance of the number of the genes declared differentially expressed can be very high for multiple testing procedures even with reasonably large sample sizes. Whenever this is the case, the stability of membership in the list of candidate genes should be expected to be low. However, the reverse is not true. If the variance of the total number of selected genes is low, there still can be tangible variations in the stability of selection for individual genes, thereby affecting the composition of the resultant list of candidate genes. This obviously can have a strong effect on the ranking of candidate genes based on purely statistical criteria.

The present study demonstrates that the proportion of highly stable (with frequencies of more than 80%) genes appears to be almost the same for all the selection procedures under study. At the same time, the overall stability of gene selection varies among different methods. The Cramér-von Mises seems to be superior to other methods in this respect. It is difficult to control the stability of gene selection by an additional adjustment of *p*-values. Indeed, for the FWER-controlling procedures, the relationship between the original (adjusted for multiple testing) *p*-values and the selection frequency appears to be non-linear. However, resampling techniques represent a universal tool for assessing the stability in question with the data at hand. As was emphasized in Section 1, our simulation studies were designed to demonstrate the fact that the correlation between gene expression levels can affect the results of testing two-sample marginal hypotheses. The FDR-controlling procedures appear to be especially sensitive to this effect. Our recent study [28] pinpoints specific components of the empirical Bayes methodology where this effect manifests itself. The quantitative contribution of the correlation between gene expression levels to the outcomes of microarray data analysis is diffcult to estimate because no tools are available to model the actual correlation structure of such a large number of variables in computer simulations.

Tables 2 and 3 also illustrate the importance of sample size. However, the number of genes selected by the Benjamini-Hochberg and nonparametric empirical Bayes procedures is very sensitive to correlations even when the power of these methods reaches 100%. The variance of the true FDR is also quite high for such procedures. Our simulations show that the FWER-controlling procedures are more stable to the effect of correlation and this stability increases with increasing sample size. Pavlidis et al. [3] proposed to approach the sample size problem from the stability perspective and we find their idea very promising and deserving of further exploration.

The distribution-free methods are generally more stable than the *t*-test. It is our firm belief that such methods will play an increasingly important role and gradually replace the *t*-test in microarray studies. Robust versions of two-sample tests in general and of the *t*-test [29] in particular can be quite competitive with distribution-free methods [30] and this avenue invites a special investigation.

## Conclusion

As larger sets of microarray gene expression data become more readily available, the stability of gene selection is becoming easier to assess using resampling techniques. We have found that some genes are selected much less frequently (across subsamples) than other genes with the same adjusted *p*-values. The relationship between the stability of gene selection and the original (adjusted) *p*-values may be rather complex but resampling techniques can advantageously be used to select the most stable genes. Using these techniques, it is also possible to assess variability of the number of selected genes. In reference to the latter indicator, all the selection procedures studied in the present paper appear to be highly unstable. For the FWER-controlling procedures, this property correlates well with the level of random fluctuations in the estimated power of a given procedure. The more conservative FWER-controlling procedures appear to be more stable to the effect of correlation than the FDR-based procedures. The stability characteristics discussed in this paper provide an additional information that should be utilized in gene selection procedures. We suggest that resampling techniques be routinely used for selection of individual genes whenever the sample size is not prohibitively small.

## Authors' contributions

The basic idea behind this study emerged from discussions between AY and AG. The detailed study design was developed by all the members of the research team. YX and XQ carried out the needed computations and simulations.

## Supplementary Material

Additional File 1 includes the executable programs employed in this study Click here for file

Additional File 2 four figures representing histograms for the number of selected genes pertaining to the simulation studies reported in Section 2.2. Click here for file
