# Supplementary material for: Assessing stability of gene selection in microarray data analysis
Source: BMC Bioinformatics. 2006 Feb 1;7:50. doi: 10.1186/1471-2105-7-50 (PMC1403808; doi:10.1186/1471-2105-7-50)
Supplement: Additional File 2 — four figures representing histograms for the number of selected genes pertaining to the simulation studies reported in Section 2.2. [file 1471-2105-7-50-S2.pdf]

Assessing Stability of Gene Selection in Microarray Data Analysis

SIMULDIST

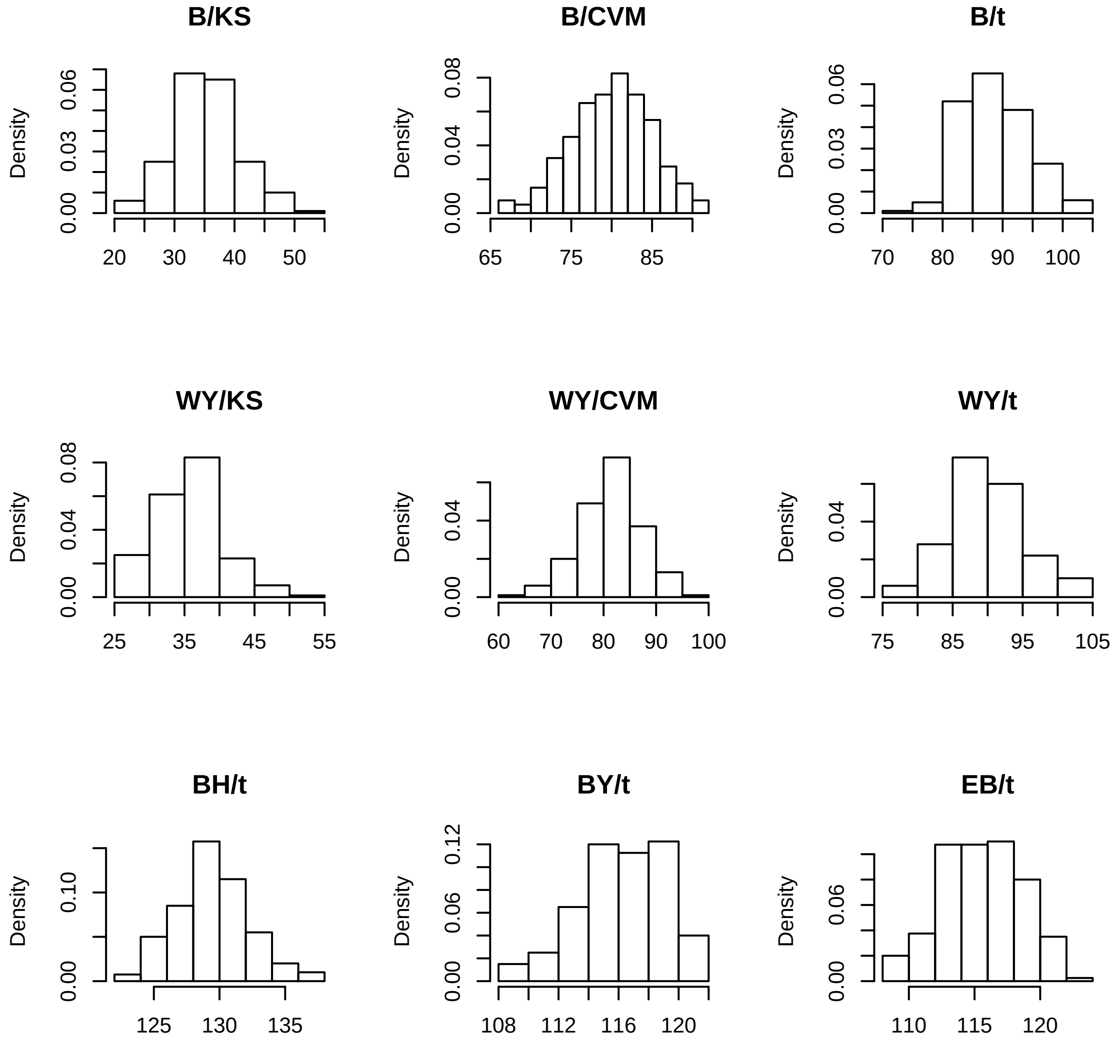

Figure 1: Histograms of the number of selected genes across 200 simulation runs constructed for S15.

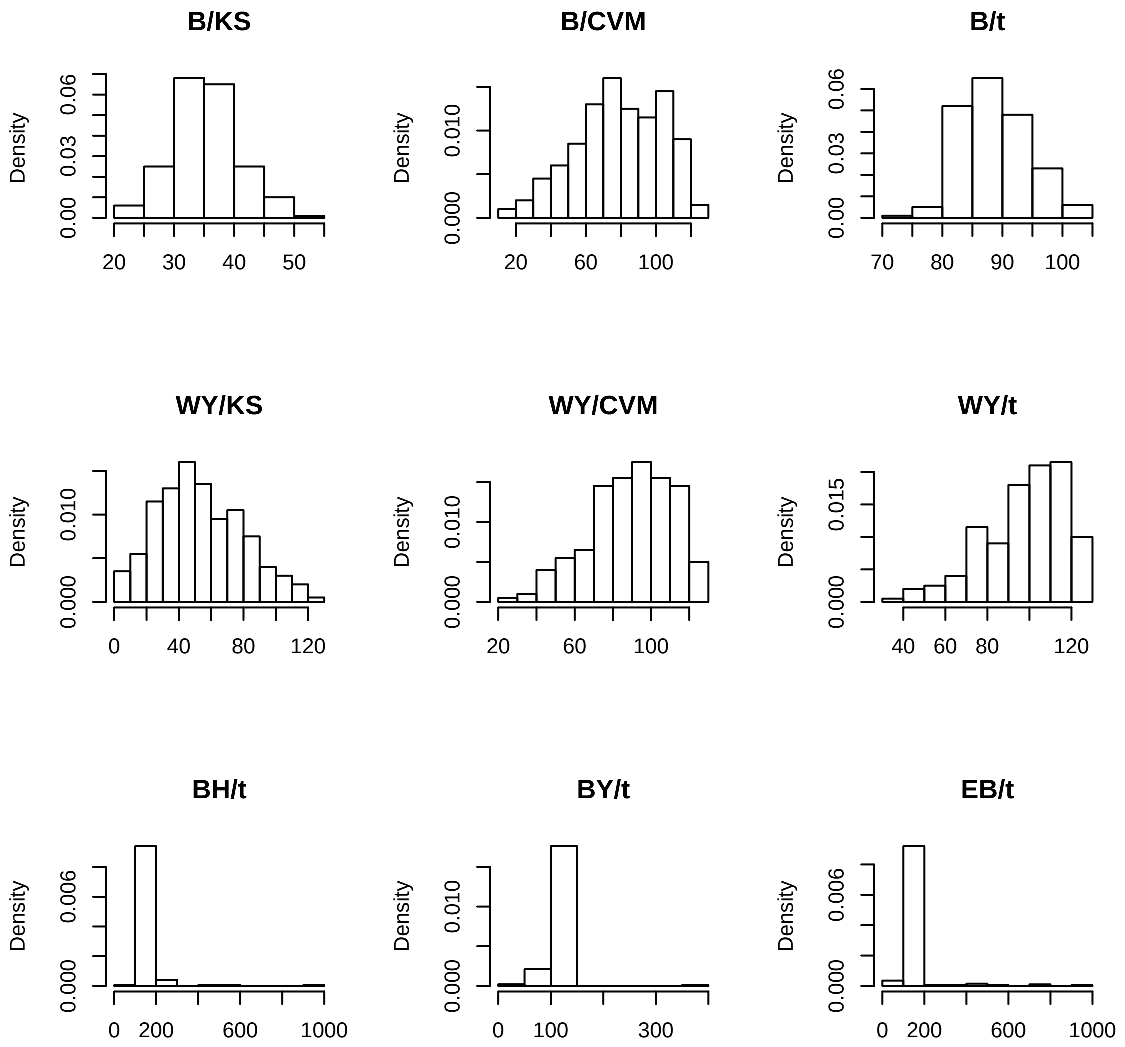

Figure 2: Histograms of the number of selected genes across 200 simulation runs constructed for S15COR.

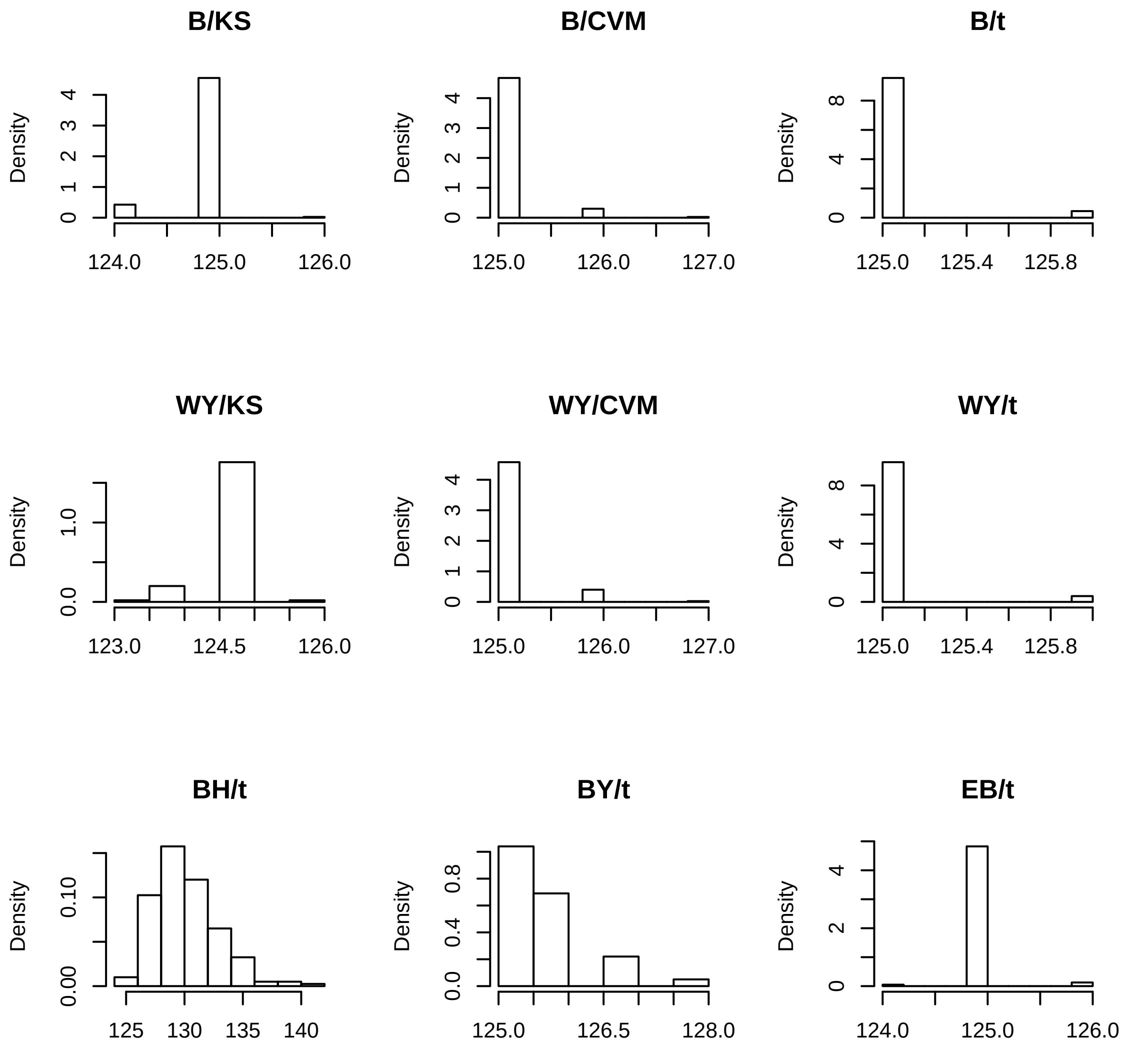

Figure 3: Histograms of the number of selected genes across 200 simulation runs constructed for S43.

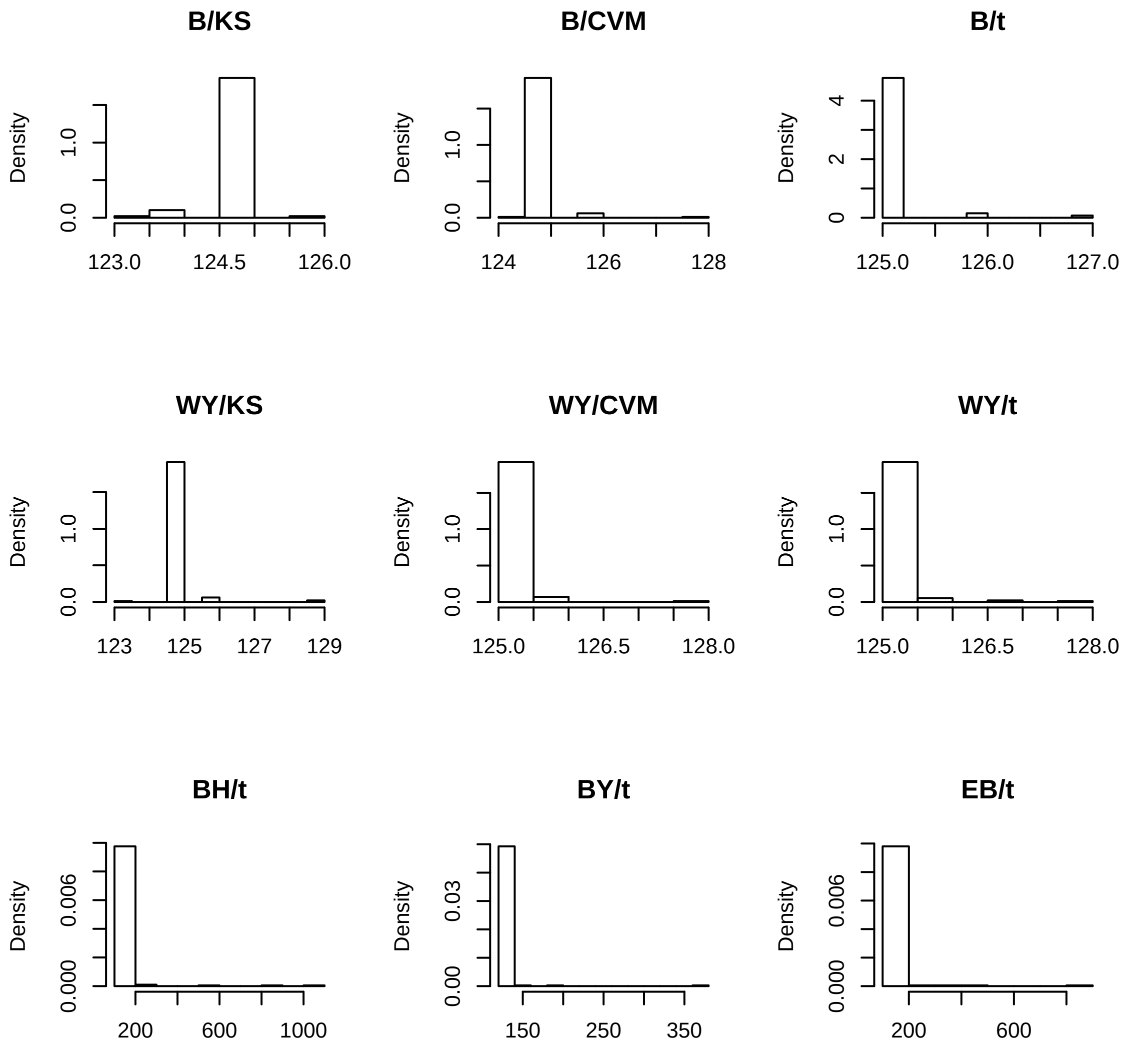

Figure 4: Histograms of the number of selected genes across 200 simulation runs constructed for S43COR.
